# Influence of Trace Elements Mixture on Bacterial Diversity and Fermentation Characteristics of Liquid Diet Fermented with Probiotics under Air-Tight Condition

**DOI:** 10.1371/journal.pone.0114218

**Published:** 2014-12-08

**Authors:** Yuyong He, Zhiyu Chen, Xiaolan Liu, Chengwei Wang, Wei Lu

**Affiliations:** 1 Jiangxi Province Key Laboratory of Animal Nutrition, Jiangxi Agricultural University, Nanchang, People’s Republic of China; 2 College of Life Science, Jiangxi Science & Technology Normal University, Nanchang, People’s Republic of China; University of Lleida, Spain

## Abstract

Cu^2+^, Zn^2+^, Fe^2+^ and I^−^ are often supplemented to the diet of suckling and early weaning piglets, but little information is available regarding the effects of different Cu^2+^, Zn^2+^, Fe^2+^ and I^−^ mixtures on bacteria growth, diversity and fermentation characteristics of fermented liquid diet for piglets. Pyrosequencing was performed to investigate the effect of Cu^2+^, Zn^2+^, Fe^2+^ and I^−^ mixtures on the diversity, growth and fermentation characteristics of bacteria in the liquid diet fermented with *Bacillus subtilis* and *Enterococcus faecalis* under air-tight condition. Results showed that the mixtures of Cu^2+^, Zn^2+^, Fe^2+^ and I^−^ at different concentrations promoted Bacillus growth, increased bacterial diversity and lactic acid production and lowered pH to about 5. The importance of Cu^2+^, Zn^2+^, Fe^2+^ and I^−^ is different for Bacillus growth with the order Zn^2+^> Fe^2+^>Cu^2+^> I^−^ in a 21-d fermentation and Cu^2+^>I^−^>Fe^2+^>Zn^2+^ in a 42-d fermentation. Cu^2+^, Zn^2+^, Fe^2+^ and I^−^ is recommended at a level of 150, 60, 150 and 0.6 mg/kg respectively for the production of fermented liquid diet with *Bacillus subtilis*. The findings improve our understanding of the influence of trace elements on liquid diet fermentation with probiotics and support the proper use of trace elements in the production of fermented liquid diet for piglets.

## Introduction

Feeding animals a non-liquid diet with probiotics can improve their intestinal health [1]–[6] and performance [7]–[9], especially with a desirable fermented liquid diet, because it can offer animals more beneficial bioactive substances including lactic acid [10], [11]. Lactic acid is known to exhibit a strong bactericidal effect towards enterobacteria under low pH conditions [12], [13]. Feeding suckling and early weaned piglets with fermented liquid diet not only keeps them a high and regular feed and water intake [14]–[17] but also alleviates the stress associated with dietary change, reduces the number of undesirable enterobacteria [18]–[21] and improves the function of intestinal mucosa [22], [23].

Nutrients including trace elements, such as Cu^2+^, Zn^2+^, Fe^2+^ and I^−^ influence the growth and metabolism of probiotics. Low or high concentration of these trace elements would be harmful to the growth of bacteria [24], [25]. Limited information is available about the effect of trace element mixtures on the growth and metabolism of probiotics in the process of liquid diet fermentation.

The purpose of this study was, therefore to investigate the effect of Cu^2+^, Zn^2+^, Fe^2+^ and I^−^ mixtures at different concentrations on the diversity, growth and fermentation characteristics of bacteria in the liquid diet fermented with probiotics under air-tight condition, and to produce high quality fermented liquid diet with the proper use of trace elements.

## Materials and Methods

### Experimental design

A basal diet was produced with the following formula: Corn 52.0%, Wheat bran 7.0%, Extruded soybean 30.0%, Fishmeal 3.0%, Lactose 4.0% and Premix 4.0%, per kg premix provides: VA 450 000 IU, VD_3_ 72 000 IU, VE 2 750 IU, VK_3_ 100 mg, VB_1_ 90 mg, VB_2_ 280 mg, VB_6_ 190 mg, VB_12_ 0.8 mg, Niacin 1 450 mg, Pantothenic acid 950 mg, Biotin 3 mg, Choline chloride 10 500 mg, Lysine 40 000 mg, Mn 2 000 mg, Co 38 mg, Se 10.5 mg, Ca 137 000 mg, P 40 800 mg, NaCl 80 000 mg. Commercial probiotics products-Wole200 (*Bacillus subtilis* HEW-D113, effective live bacteria ≥2×10^10^CFU/g) and Wosun100 (*Enterococcus faecalis* HEW-A208, effective live bacteria ≥1×10^10^CFU/g) that produced by Beijing Heswof biotechnology CO., LTD (China) were supplemented to the basal diet at a level of 300 mg/kg, respectively. The nutrient levels (analyzed value except digestible energy) of the basal diet are as follows (on air-dry basis): Digestible energy (Calculated) 13.71 MJ/kg, Crude protein 19.75%, Calcium 1.05%, Total phosphorus 0.66%, Lysine 1.32% and Methionine plus Cystine 0.78%.

Cu^2+^ (uSO_4_•5H_2_O), Zn^2+^ (ZnSO_4_•H_2_O), Fe^2+^ (FeSO_4_•H_2_O) and I^−^ (KI) were selected as experimental factors, each factor had three levels (Table 1). L_9_ (3^4^) Orthogonal design (Table 2) was performed to optimize factor level and their combination.

**Table 1:** Levels of different ions that added to basal diet (mg/kg basal diet).

	Level 1	Level 2	Level 3
Cu^2+^	200	150	100
Zn^2+^	160	110	60
Fe^2+^	150	100	50
I^−^	2.4	1.2	0.6

**Table 2:** L_9_ (3^4^) Orthogonal experiment (mg/kg basal diet).

Treatment No.	Cu^2+^	Zn^2+^	Fe^2+^	I^−^
1	200	160	150	2.4
2	200	110	100	1.2
3	200	60	50	0.6
4	150	160	100	0.6
5	150	110	50	2.4
6	150	60	150	1.2
7	100	160	50	1.2
8	100	110	150	0.6
9	100	60	100	2.4

### Preparation and sampling

According to Table 2, nine mineral mixtures were prepared, then each mineral mixture was mixed with basal diet and commercial probiotics products to produce experimental diet.

100 g experimental diet and 300 g tap water was placed in each polypropylene bag (size 18 cm×15 cm, thickness 80 µm) with a total of 20 bags in each treatment, air in the bag was removed artificially before the bag heat-sealed, all bags were loaded into a steaming box immediately after a two-hour fermentation and cooked with steam at 80°C for 30 min under normal pressure, then removed from the box and fermented at room temperature varied from 22.5°C to 33.9°C in summer. Samples from nine treatments on day 0, 21 and 42 were marked as A1–A9, B1–B9 and C1–C9, respectively. Four replicate samples from each treatment were collected on each time point, respectively for pH measurement, lactic acid determination and bacterial genomic DNA extraction.

### DNA extraction, PCR, Amplicon quantitation and Pyrosequencing

Bacterial genomic DNA from each sample was extracted using the E.Z.N.A Soil DNA kit (OMEGA, USA) and the triplicate DNA extracts for each sample were pooled prior to PCR. PCR amplification covering the V1–V3 region of the 16S rRNA bacterial gene was performed to construct community library through tag pyrosequencing. The bar-coded primers 27F and 533R containing A and B sequencing adaptors (454 Life Sciences) were used. The forward primer (B-27F) was 5′-*CCTATCCCCTGTGTGCCTTGGCAGTCGACT*AGAGTTTGATCCTGGCTCAG-3′, where the sequence of the B adaptor is shown in italics and underline, the reverse primer (A-533R) was 5′- *CCATCTCATCCCTGCGTGTCTCCGACGACT*NNNNNNNNNNTTACCGCGGCTGCTGGCAC-3′, where the sequence of the A adaptor is shown in italics and underlined and the Ns represent a ten-base sample specific barcode sequence [26].

The PCRs were carried out in a 20 mL reaction volume containing 0.4 µl TransStart Fastpfu DNA Polymerase (Beijing TransGen Biotech Co., Ltd, China), 4 µl 5×FastPfu buffer, 2 µl 2.5 mM dNTPs, 0.8 µl 5 µM Forward Primer, 0.8 µl 5 µM Reverse Primer, 0.4 µl 5 µM Fastpfu Polymerase, 10 ng DNA template and de-ionized ultrapure water. PCR protocol was performed on ABI GeneAmp 9700Cycler using the following conditions: initial denaturation for 2 min at 95°C, followed by 25 cycles of denaturation for 30 s at 95°C, annealing for 30 s at 55°C and extension for 30 s at 72°C, then, with a final extension for 5 min at 72°C. Amplification products were visualized on 2% agarose gels, then purified using AxyPrepDNA PCR purification kit (Axygen, China), quantified using the QuantiFluor-ST system (Promega) and pooled in equimolar ratios based on concentration and subjected to emulsion PCR (Roche GS FLX Titanium emPCR Kits) to generate amplicon libraries, Amplicon pyrosequencing was performed from the A-end using a 454/Roche GS-FLX Titanium platform at Majorbio Bio-Pharm Technology Co., Ltd., Shanghai, China.

### Sequences processing and Bioinformatic analysis

Raw sequences obtained from 454/Roche GS-FLX Titanium pyrosequencer were processed with Mothur software (http://sourceforge.net/projects/seqclean/& http://www.mothur.org/wiki/Main_Page) and the unqualified sequences were removed according to the following criteria: <200 nucleotides in length (not including sample specific barcodes), contained ambiguous bases, had an imperfect match to a sample-specific barcode and a read quality score <25. The chimeric sequences were also excluded by usearch (version6.1, http://drive5.com/usearch/). The unique sequences were clustered into operational taxonomic units (OTUs) at 97% sequence identity by using Qiime software (http://qiime.org/scripts/assign_taxonomy.html, Naïve Bayesian Classifier). Taxonomy assignment was conducted using silva of bacterial ribosomal database (version115, http://www.arb-silva.de) with a confidence level of 0.7. The abundance coverage-based estimator (ACE, http://www.mothur.org/wiki/Ace) and Shannon index (http://www.mothur.org/wiki/Shannon) were calculated by Mothur, Mcrobial community barplot and Heatmap were generated by R packages.

### Lactic acid determination and pH measurement

Lactic acid production of fermented liquid diets was determined by using D-/L- lactic acid test kits (Nanjing Jiancheng Bioengineering Institute, China), and the pH of each sample was measured using a digital pH meter (LP115FK, China) after calibration with standard buffers of pH 4.0 and 7.0.

### Statistical analysis

Statistical tests were performed using one-way ANOVA (SAS, 2004) followed by Duncan’s test. Bars indicate ±standard deviation of the mean (n = 4). Within the same group, columns with the same letter are not statistically different (*P*>005) and with the different small letters are statistically different (*P*<0.05).

## Results

### Data Summary of Pyrosequencing

After stringent quality assessment and data filtering, high quality reads produced in this experiment have been deposited in NCBI database (accession number: SRP044186). A total of 372614 valid reads and 32982 OTUs were obtained from the 27 samples including 147166 reads and 7184 OTUs in A libraries, 123683 reads and 13463 OTUs in B libraries, 101765 reads and 12335 OTUs in C libraries, respectively. Each sample contains 14988 to 18572 reads with OTUs ranging from 668 to 902 in A libraries, 8584 to 18604 reads with OTUs ranging from 1082 to 1671 in B libraries and 8842 to 14028 reads with OTUs ranging from 1161 to 1539 in C libraries, respectively.

### Bacterial community Diversity

**Figure 1:**
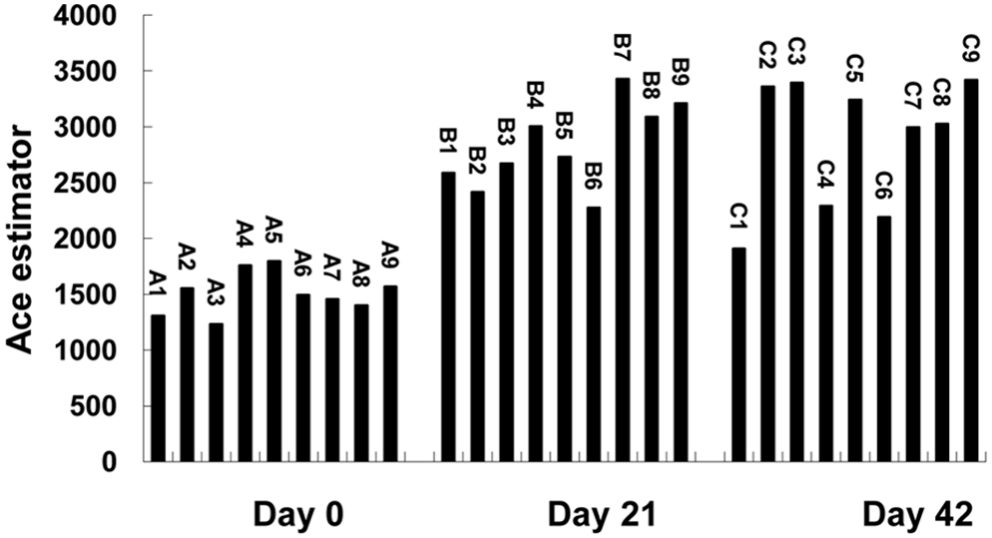
Ace estimator of each treatment in different fermentation time.

**Figure 2:**
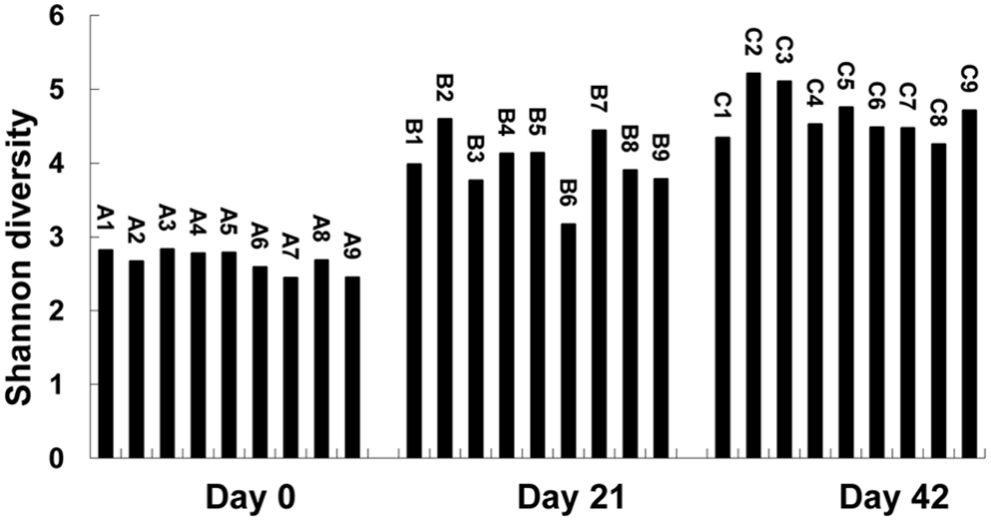
Shannon diversity of each treatment in different fermentation time.

The Ace of each treatment on day 21 and 42 was higher than that on day 0, respectively, and the Ace estimator of treatment 2, 3, 5 and 9 constantly increased from day 0 to day 42 (Figure 1). Shannon index of each treatment also increased with the advancement of fermentation (Figure 2).

### Microbial community barplot

**Figure 3:**
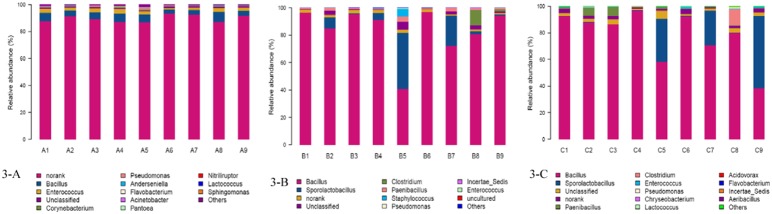
Genus-level taxonomic compositions. Genus-level taxonomic compositions of fermented liquid diet on day 0 (A libraries), day 21 (B libraries) and day 42 (C libraries). Sequences that could not be classified into any known group were assigned as Norank, sequences that could not be matched to any known sequences were designated as unclassified, sequences that had relative abundance of less than 1% were grouped into others.

Nine libraries from A1 to A9 showed almost the similar 16S rRNA profiles in genus level distributions (Figure 3-A), *Bacillus* and *Enterococcus* had a low relative abundance, represented 2.84%–7.52% and 1.10%–3.33% of the reads, respectively, the most abundant genus was Nonrank which accounted for 86.94%–93.54% of reads. With the advancement of fermentation, B libraries (Figure 3-B) were dominated by *Bacillus* with an exception in B5 library, B5 library was dominated by *Sporolactobacillus* with 41.12% reads. The relative abundance of Bacillus in B libraries accounted for 40.73%–96.89% and ranked in order B6>B1>B3>B9>B4>B2>B8>B7>B5. The relative abundance of *Enterococcus* varied from 0.00% to 0.40% in B libraries and ordered as B5>B2>B3>B9>B1>B7>B4>B6>B8. On day 42 (Figure 3-C), excepted for C9 library, the other C libraries contained the most abundant *Bacillus*, which represented 58.28%–96.71% of the reads and the relative abundance of Bacillus ordered as C4>C1>C6>C2>C3>CC8>C7>C5, C9 library was numerically dominated by *Sporolactobacillus* with 54.33% of the reads. The relative abundance of *Enterococcus in* C libraries accounted for 0.14%–1.15% and sequenced as C6>C9>C8>C2>C5>C3>C1>C4>C7.

### Heatmap analysis

**Figure 4:**
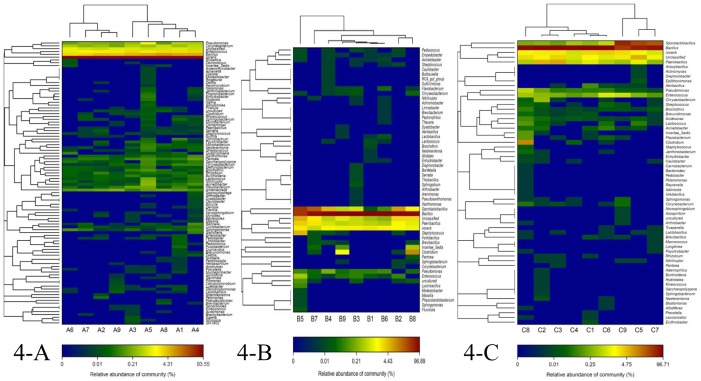
Hierarchical dendrogram of bacterial distribution. Bacterial distribution of the top 100 abundant genus in the fermented liquid diet that sampled from A libraries, B libraries and C libraries. Double hierarchical dendrogram shows the bacterial distribution. The bacterial phylogenetic tree was calculated using the neighbor-joining method and the relationship among samples was determined by Bray-Curtis distance. The heatmap plot depicts the relative percentage of each bacterial genus within each sample. The relative values for bacterial genus are indicated by color intensity with the legend at the bottom.

The hierarchical heatmap (Figure 4) is based on the top 100 abundant bacterial community at genus level, and the heatmap constructed with samples from A libraries (Figure 4-A) disclosed that A libraries was numerically dominated by Nonrank genus, followed by *Bacillus* and *Enterococcus*, the highest similarity of the libraries could be found in A2 and A9 library, A1 and A4 library, respectively. A2 and A9 library grouped together firstly and then clustered with A7 and A6 library in order, A1 and A4 library clustered together and grouped with A8, A5 and A3 library in order. On day 21 (Figure 4-B), B libraries had the highest relative abundance of Bacillus with an exception in B5 library, so, B5 library grouped alone and the other B libraries clustered together, the highest similarity of the libraries existed in B1 and B6 library, B2 and B8 library, respectively. On day 42 (Figure 4-C), C5, C7 and C9 library clustered together owing to this three libraries contained higher relative abundance of Sporolactobacillus than the other C libraries, the highest similarity of the libraries was found in C5 and C7 library, C2 and C3 library, C1 and C6 library, respectively.

### Lactic acid production and pH

**Figure 5:**
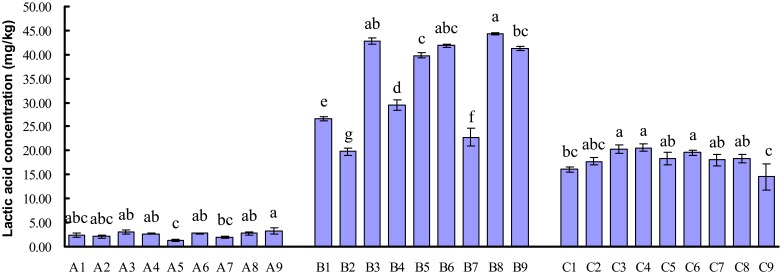
Lactic acid production of different fermented liquid diets on day 0, 21 and 42.

**Figure 6:**
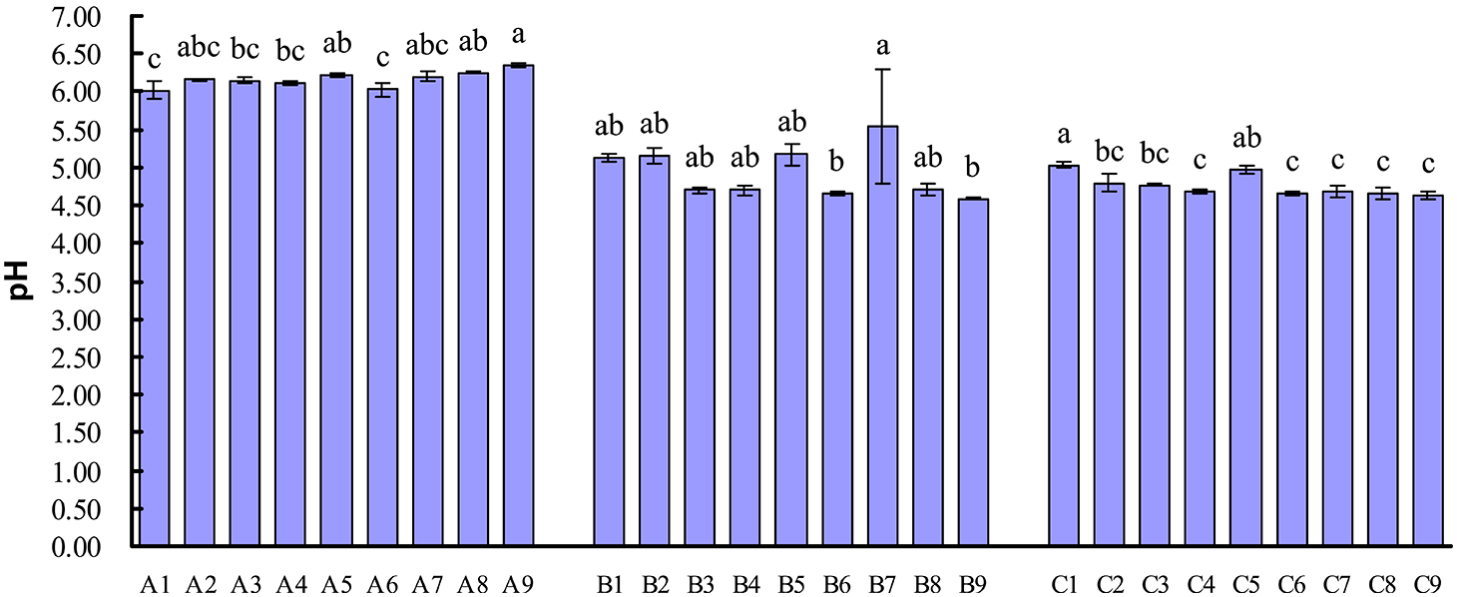
pH of different fermented liquid diets on day 0, 21 and 42.

As shown in Figure 5, the concentration of lactic acid in samples from C treatments (day 42) was higher than that in samples from A treatments (day 0), but lower than that in samples from B treatments (day 21). Lactic acid content of samples in A treatments changed from 1.32 mg/kg to 3.23 mg/kg, sample in A9 had higher (*P*<0.05) lactic acid concentration than sample in A5 and A7, respectively. The content of lactic acid in B treatments varied from the minimum of 19.85 mg/kg in B2 sample to the maximum of 44.28 mg/kg in B8 sample and ranked as B8>B3>B6>B9>B5>B4>B1>B7>B2, the level of lactic acid in B3, B5, B6, B8 and B9 was higher (P<0.05) than that in B1, B2, B4 and B7, respectively. In C treatments, the concentration of lactic acid changed from the minimum of 14.57 mg/kg in C9 sample to the maximum of 20.61 mg/kg in C4 sample and ordered as C4>C3>C6>C8>C5>C7>C2>C1>C9, there was no significant difference (*P*>0.05) in lactic acid concentration among C2, C3, C4, C5, C6, C7 and C8. pH in samples from B and C treatments was lower than that of samples from A treatments (Figure 6), respectively. pH in samples from B treatments changed from 4.59 to 5.15 and sequenced as B7>B5>B2>B1>B8>B3 = B4>B6>B9, there was no significant difference (*P*>0.05) in pH value among B1, B2, B3, B4, B5, B6, B8 and B9. Samples from C treatments had a pH from 4.64 to 5.05 and ordered as C1>C5>C2>C3>C4 = C7>C6 = C8>C9, the level of pH in samples among C2, C3, C4, C6, C7, C8 and C9 showed no significant difference (*P*>0.05).

### Comprehensive analysis of orthogonal experiment results

Zn^2+^ was the most important ion in controlling Bacillus growth, lactic acid concentration and pH of fermented liquid diet during a 21-d fermentation (Table 3), elevated Zn^2+^ level increased lactic acid concentration and lowered pH. Cu^2+^ played a dominant role in Bacillus growth and pH of fermented liquid diet during a 42-d fermentation (Table 4). Cu^2+^, Zn^2+^, Fe^2+^ and I^−^ would be recommended at a level of 150, 60, 150 and 0.6 mg/kg respectively to achieve ideal Bacillus growth, lactic acid production and pH when fermented liquid diet with *Bacillus subtilis*.

**Table 3:** Comprehensive analysis of Orthogonal experiment results on day 21.

Items	Factors	Mean (B treatment)			Range (R)	Best level	Rank
		Level 1	Level 2	Level 3			
Bacillus	Cu^2+^	92.31	76.23	82.51	16.08	1	Zn^2+^>Fe^2+^>Cu^2+^>I^−^
	Zn^2+^	86.57	68.92	95.56	26.64	3	
	Fe^2+^	91.43	90.23	69.39	22.04	1	
	I^−^	77.24	84.72	89.09	11.85	3	
Enterococcus	Cu^2+^	0.05	0.14	0.02	0.12	2	Fe^2+^>Zn^2+^ = I^−^>Cu^2+^
	Zn^2+^	0.02	0.16	0.04	0.14	2	
	Fe^2+^	0.01	0.04	0.16	0.15	3	
	I^−^	0.16	0.04	0.02	0.14	1	
Lactic acid	Cu^2+^	29.75	37.02	36.12	7.27	2	Zn^2+^>I^−^>Fe^2+^>Cu^2+^
	Zn^2+^	26.28	34.66	41.95	15.67	3	
	Fe^2+^	37.58	30.17	35.14	7.42	1	
	I^−^	35.91	28.17	38.82	10.65	3	
pH	Cu^2+^	4.99	4.84	4.95	0.15	2	Zn^2+^>I^−^>Fe^2+^>Cu^2+^
	Zn^2+^	5.13	5.01	4.65	0.48	3	
	Fe^2+^	4.83	4.81	5.14	0.33	2	
	I^−^	4.96	5.12	4.70	0.42	3	

**Table 4:** Comprehensive analysis of Orthogonal experiment results on day 42.

Items	Factors	Mean (C treatment)			Range (R)	Best level	Rank
		Level 1	Level 2	Level 3			
Bacillus	Cu^2+^	88.92	82.42	63.14	25.78	1	Cu^2+^>I^−^>Fe^2+^>Zn^2+^
	Zn^2+^	86.67	75.45	72.36	14.31	1	
	Fe^2+^	88.31	74.38	71.80	16.52	1	
	I^−^	63.10	83.67	87.72	24.63	3	
Enterococcus	Cu^2+^	0.27	0.53	0.48	0.26	2	Zn^2+^>Fe^2+^>Cu^2+^>I^−^
	Zn^2+^	0.18	0.35	0.75	0.57	3	
	Fe^2+^	0.62	0.44	0.23	0.39	1	
	I^−^	0.46	0.53	0.29	0.24	2	
Lactic acid	Cu^2+^	18.06	19.50	16.99	2.50	2	I^−^>Cu^2+^>Fe^2+^>Zn^2+^
	Zn^2+^	18.24	18.14	18.17	0.10	1	
	Fe^2+^	18.03	17.65	18.88	1.23	3	
	I^−^	16.32	18.46	19.77	3.45	3	
pH	Cu^2+^	4.98	4.77	4.66	0.32	3	Cu^2+^>I^−^>Fe^2+^>Zn^2+^
	Zn^2+^	4.85	4.81	4.76	0.09	3	
	Fe^2+^	4.83	4.71	4.88	0.17	2	
	I^−^	4.92	4.72	4.78	0.20	2	

## Discussion

In order to gelatinize corn starch and sterilize some undesirable microorganism, liquid diet which inoculated with *Bacillus subtilis* and *Enterococcus faecalis* was packed with plastic bag, then put into a box and heated with steam at 80°C for 30 minutes prior to fermentation, data in Figure 3 indicated that the relative abundance of Bacillus in all treatments on day 21 and 42 was respectively higher than that on day 0, however, the relative abundance of Enterococcus on day 21 and 42 was respectively lower than that on day 0, this indicated that Bacillus is more resistant to high temperature than Enterococcus and this is similar to the previous results [27]–[29]. The growth check of Enterococcus in 9 treatments was also possibly related to the production of antibacterial peptide in the process of fermentation and the concentration of trace elements involved, Hyronimus et al. (1998) found that Enterococcus bacteria was killed by the antibacterial peptide produced by Bacillus [30], further work is needed to clarify the factors that decreased Enterococcus growth.

The intracellular concentrations of trace elements ion must be finely adjusted to avoid either deprivation or toxicity and careful homeostasis is very important for the optimal growth of microbia. Many concerns are focus on the effect of mixed trace elements on microbial growth and its metabolism. Results in this experiment showed that the relative abundance of Bacillus in different treatments was different, this implicated that Bacillus growth was influenced by the mixture of Cu^2+^, Zn^2+^, Fe^2+^ and I^−^ at different concentration levels.

Otludil et al. (2005) confirmed that the growth of *Bacillus subtilis* was inhibited strongly by Cu^2+^ at a high level [31], Rathnayake et al. (2010) and Lee et al. (2011) also demonstrated *Bacillus thuringeinsis* were highly sensitive to Cu^2+^ compared to Zn^2+^ [32], [33], however, in this experiment Bacillus had a higher abundance at high divalent copper concentration than at a low concentration, this probably due to this concentration of divalent copper involved is not toxic to Bacillus growth or the toxicity of divalent copper at 200 mg/kg was alleviated by divalent zinc, because zinc ion can displace copper ion from site-specific loci and low the production of reactive oxygen species including hydroxyl radicals, hydrogen peroxide and superoxide [34], [35]. Zinc starvation causes cells to die, but when the extracellular concentration of zinc exceeds the capacity of the zinc homeostasis, it becomes cytotoxic and enhanced intracellular zinc concentration triggers apoptosis [36], [37]. Ali et al. (2012) found that when the concentration of Zn^2+^ increased from 25 to 200 mg/ml, the growth of Bacillus firstly increased within a normal concentration and then decreased in an excessive dose [38]. In this study, all the involved divalent zinc concentrations are not toxic to the growth of Bacillus in the fermented liquid diet at day 42 (Table 3), but there is an exception in the fermented liquid diet at day 21, this is probably caused by the cytotoxicty in a 21-d incubation and the adaptation in a 42-d fermentation, and further study is needed to explore the reasons for this findings.

Boyaval (1989) found that Fe had a stimulatory effect on bacterial growth within a certain concentrations [39], but bivalent Fe is unstable in aqueous media and excessive Fe^2+^ tends to react with molecular oxygen to form Fe^3+^ and superoxide with a consequence of repressing microbial growth by lipid peroxidation and DNA damage [37]. Data in Table 2 and 3 indicate that 150 mg/kg Fe^2+^ is not a toxic dose and 50 mg/kg Fe^2+^ is insufficient for Bacillus growth during a 21 or 42-d continuous fermentation. Excessive iodine can decrease microbial biogass or abundance by inhibiting the growth and viability of bacteria [40] or by degrading the spore coat protein of Bacillus subtilis [41], this is further validated by the results in Table 3 and 4, the abundance of Bacillus genus in the fermented liquid diet supplemented iodine at 2.4 mg/kg was much lower than that at 1.2 mg/kg or 0.6 mg/kg.

During the process of fermentation, organic acids including lactic acid are formed as a result of bacterial growth and metabolism. Lactic acid has been used as preservative, inhibitor of bacterial spoilage, acidifying agent or flavouring substance in food and feed industry [42]. Although there are numerous reports on lactic acid production by some Bacillus species including *Bacillus coagulans* [43], [44] and *Bacillus subtilis* [45]–[47], little work has been done to evaluate the combined effects of Cu^2+^, Zn^2+^, Fe^2+^ and I^−^ ion on lactic acid content produced by Bacillus. As shown in Table 3 and 4, Cu^2+^, Zn^2+^, Fe^2+^ and I^−^ ion had an important effect on lactic acid production and pH of fermented liquid diet on day 21. Low concentration of Zn^2+^ and I^−^ promoted Bacillus growth, thus resulting in increasing in lactic acid production. High Fe^2+^ and low Cu^2+^ concentration in the fermented liquid diet had a positive effect in increasing lactic acid production and lowering pH, which is consistent with the results observed by Boyaval (1989) that Fe had a stimulatory effect on lactic acid production [39].

The lactic acid production of fermented liquid diet on day 42 decreased compared with that on day 21, the pH of all samples on day 42 was also lower than that on day 21 excluded C3 and C9. This phenomenon might be explained by the shift of production of organic acid from lactic acid to other organic acids (propionic acid, butyric acid, et al.) and by the formation of other organic acids with lactic acid as a substrate. Some authors reported that when the pH value of fermentation media dropped to 5–4, the growth of lactic acid bacteria and the lactic acid production decreased [42], [48].

## Conclusion

Bacillus concentration, bacterial diversity, lactic acid level and pH in the fermented liquid diet supplemented with *Bacillus subtilis* varies with mixtures of minerals at different concentration levels. *Enterococcus faecalis* is sensitive to high temperature (80°C) and the practice of heating liquid diet with *Enterococcus faecalis* together prior to fermentation is not suggested. High Bacillus growth and lactic acid concentration and low pH can be achieved in a fermented liquid diet When supplemented minerals mixture (Cu^2+^ 150 mg/kg, Zn^2+^ 60 mg/kg, Fe^2+^ 150 mg/kg and I^−^ 0.6 mg/kg) and *Bacillus subtilis* (300 mg/kg) to a liquid diet (corn-extruded soybean based diet∶tape water = 1∶3) for a 21-d fermentation.
